# Levels of anxiety, depression and stress among health care workers during the COVID19 pandemic: Study conducted at the University Hospital Farhat Hached of Sousse-Tunisia

**DOI:** 10.1192/j.eurpsy.2023.1728

**Published:** 2023-07-19

**Authors:** N. Belhadj Chabbah, S. Chatti, Z. Athimni, M. Bouhoula, A. Chouchane, A. Aloui, I. Kacem, M. Maoua, A. Brahem, H. Kalboussi, O. Elmaalel, N. Mrizek

**Affiliations:** Department of Occupational Medicine, Farhat Hached” University Hospital Center, Sousse, Tunisia

## Abstract

**Introduction:**

During the COVID-19 pandemic, health care workers found themselves threatened by developing psychological effects.

**Objectives:**

The objective of this study is to evaluate the impact of exposure to COVID-19 on the mental health of medical and paramedical staff at Farhat Hached Hospital in Sousse and to identify potential risk factors.

**Methods:**

This is a descriptive cross-sectional study that included 166 health care workers of Farhat Hached Hospital of Sousse throughout 3 months. The patient health questionnaire (PHQ-9), the generalized anxiety disorder (GAD-7), and the revised event impact scale (IES- R) were used to assess depression, anxiety, and stress respectively.

**Results:**

The mean age of the participants was 37.06 ±11.07 years with a female predominance (80.1%). The median professional seniority was 7.5 years with extremes ranging from 1 to 39 years. Nurses were the most represented (34.3%) followed by medical residents (24.7%). PHQ-9, GAD-7, and IES- R scores revealed that 51.8%, 40.4%, and 28.3% of participants had moderate or severe levels of depression, anxiety, and stress, respectively. Personal history of psychiatric disorders was significantly associated with depression (p<10-3) and anxiety (p=0.004). On the other hand, paramedical staff had a significantly higher risk of experiencing symptoms of depression (OR = 2.40 ; 95% CI [1.28-4.48] ; p= 0.006) and stress (OR = 2.03 ; 95% CI [1.01-4.11] ; p= 0.04) than medical personnel.

**Conclusions:**

This study reported a high prevalence of symptoms of anxiety, depression and stress among health care workers. Improving mental well-being and providing psychological support to health care workers is recommended.

**Disclosure of Interest:**

None Declared

